# Exploring sexual dimorphism of the modern human talus through geometric morphometric methods

**DOI:** 10.1371/journal.pone.0229255

**Published:** 2020-02-14

**Authors:** Rita Sorrentino, Maria Giovanna Belcastro, Carla Figus, Nicholas B. Stephens, Kevin Turley, William Harcourt-Smith, Timothy M. Ryan, Stefano Benazzi

**Affiliations:** 1 Department of Biological, Geological and Environmental Sciences, University of Bologna, Bologna, Italy; 2 Department of Cultural Heritage, University of Bologna, Ravenna, Italy; 3 ADES, UMR 7268 CNRS/Aix-Marseille Université/EFS, Aix-Marseille Université, Bd Pierre Dramard, France; 4 Department of Anthropology, Pennsylvania State University, State College, PA, United States of America; 5 Department of Anthropology, University of Oregon, Eugene, OR, United States of America; 6 Graduate Center, City University of New York, New York, NY, United States of America; 7 New York Consortium in Evolutionary Primatology, New York, NY, United States of America; 8 Department of Anthropology, Lehman College, New York, NY, United States of America; 9 Division of Paleontology, American Museum of Natural History, New York, NY, United States of America; 10 Department of Human Evolution, Max Planck Institute for Evolutionary Anthropology, Leipzig, Germany; The Cyprus Institute, CYPRUS

## Abstract

Sex determination is a pivotal step in forensic and bioarchaeological fields. Generally, scholars focus on metric or qualitative morphological features, but in the last few years several contributions have applied geometric-morphometric (GM) techniques to overcome limitations of traditional approaches. In this study, we explore sexual dimorphism in modern human tali from three early 20th century populations (Sassari and Bologna, Italy; New York, USA) at intra- and interspecific population levels using geometric morphometric (GM) methods. Statistical analyses were performed using shape, form, and size variables. Our results do not show significant differences in shape between males and females, either considering the pooled sample or the individual populations. Differences in talar morphology due to sexual dimorphism are mainly related to allometry, i.e. size-related changes of morphological traits. Discriminant function analysis using form space Principal Components and centroid size correctly classify between 87.7% and 97.2% of the individuals. The result is similar using the pooled sample or the individual population, except for a diminished outcome for the New York group (from 73.9% to 78.2%). Finally, a talus from the Bologna sample (not included in the previous analysis) with known sex was selected to run a virtual resection, followed by two digital reconstructions based on the mean shape of both the pooled sample and the Bologna sample, respectively. The reconstructed talus was correctly classified with a P_post_ between 99.9% and 100%, demonstrating that GM is a valuable tool to cope with fragmentary tali, which is a common occurrence in forensic and bioarchaeological contexts.

## Introduction

It is well known that males have more robust bones and a greater stature than females due to differences in genetics and developmental factors, which affect growth rates, body composition, lean muscle mass, and hormonal levels—among other things [1,2]. Accordingly, sexual dimorphism in human skeletal structures is expressed as differences in both size and shape. The accurate quantification of these phenotypic differences are fundamental to any bioarchaeological/forensic reconstruction of individual biological and/or demographic profile [3–5]. In forensic osteology, the identification of human remains is the primary focus, often requiring investigators to build a biological profile of one or more deceased individuals [6–9]. Often, this serves a humanitarian need in providing closure, as deaths occur under natural, criminal, and catastrophic circumstances [8,10–14]. One of the crucial aspects of forming a biologic profile is the estimation of an individual’s sex, being that this characteristic informs many other quantitative estimations (e.g. age, ancestry, and stature) [3,7,15,16]. Furthermore, sex assessment reduces the possible matches by half [17] and is of great importance in paleodemography and paleopathology [18].

When the entire skeleton is preserved, many methods may be employed to reliably determine sex [19], but these often rely on the most dimorphic skeletal elements (i.e., pelvis and cranium) [2,3,18,20–22], with others incorporated to strengthen the attribution [1–3]. Unfortunately, skeletal remains recovered in forensic/archaeological contexts are any combination of fragmentary, incomplete, isolated, or commingled [23–25]. As a result of this, there have been new methods developed to help build accurate biological profiles from isolated, and previously non-diagnostic (i.e. not the pelvis/skull), bones [26–32]. Of these, those of the foot tend to be recovered in isolation from context with complex taphonomy, such as natural disasters [33,34]. From this, there have been a number of studies utilizing linear measurements to determine sex from the calcaneus and the talus [17,33,35–41]. While useful, this approach is limited because each measurement is taken between two points without taking into account their relationship to the series of other paired measurements [42,43]. Landmark-based geometric morphometrics (GM) has the potential to overcome this issue, because it includes simultaneously all information about all pairwise distances between the landmarks, the curvature between them, and the angles [5,28,44–47]. This is advantageous because, although talar morphology is tightly correlated with locomotor strategy [48], other factors could influence intra-specific talar variation (e.g., body mass, environmental loading, and sexual dimorphism) [37,49–51]. For instance, women and men are known to have different gait kinematics, postural supports, and joint angles [52–54]. Here we explore whether these differences are reflected in talar shape, hypothesizing that sexually dimorphic characteristics will be evident. Further, common forensic/bioarchaeological taphonomic contexts (e.g. mass disasters, crime, exposure, and postmortem burial, etc.) often result in poor skeletal preservation (e.g. fracture, fragmentation, burning, etc.), which complicates analysis [8,10,23,55–57]. In these cases, fragmentary bones with cracks and/or gaps are usually discarded from traditional analysis because they lack the morphology necessary for accurate measurement [7,23]. However, new approaches have been developed to cope with them, such as molecular analysis or virtual reconstruction [7,58–61]. With regard to the latter, geometric morphometrics offer the possibility to virtually reconstruct missing data from partially damaged specimens [59,62]. Particularly, the use of semilandmarks allows estimates of missing data from the information that is present using the thin-plate spline interpolation, ultimately allowing the use of virtually reconstructed specimens for forensic evaluation like sex determination [59,60,63].

Here we use GM to explore sexual dimorphism of the talus from osteological collections representing three modern human populations (Sassari and Bologna from Italy, and New Yorkers from USA). First, we test the hypothesis that the amount of sexual dimorphism in the talus is population specific [3]. Second, we investigate the contribution of shape, form (shape + size) and size in determining sexual dimorphism in talar morphology at the intra- and interspecific population level. Third, we use a digitally damaged talus with known sex (not included in the previous analyses) and provide a virtual reconstruction of the missing portions using GM techniques. Overall, our extensive morphometric study of the talus aims to assess the most accurate approach for sex discrimination of isolated tali, ultimately providing useful tools for forensic and archaeological investigations.

## Materials and methods

### Data collection

The tali (N = 98) collected in this study belong to three modern human groups of urban societies from the late 19th and early 20th century, for which the sex is known from cemetery and municipal records. The sample consists of 39 individuals from Bologna (18 females and 21 males), 36 individuals from Sassari (17 females and 19 males) and 23 individuals from New York (9 females and 14 males).

The identified skeletal remains (by age, sex, cause of death, occupation) of Sassari and Bologna are part of the Frassetto collection housed at the Museum of Anthropology of the University of Bologna (Italy). The Bologna sample consists of individuals from the Certosa Cemetery (Bologna, Italy) who died between 1898 and 1944 [64]. The Sassari sample (Sardinia, Italy) consists of individuals who died in the first half of the twentieth century and were exhumed from municipal cemeteries [65]. The New York sample is represented by early 20th century post-industrial individuals from New York (USA) stored at the Smithsonian Museum of Natural History (Washington DC, USA) [66]. Specimen numbers and sex of the individuals under study are provided in S1 Appendix.

As there is a strong degree of symmetry in non-pathological human talus [67], left tali (N = 96) were preferred in the selection for the analysis. In cases where the left talus was absent or damaged (either fragmentary or affected by pathological conditions), the right one (N = 2) was selected and the digital model (see below) was mirrored.

Three-dimensional (3D) digital models of each bone were obtained either by computed tomography (CT) or surface laser scanning, as recent contributions confirmed comparable results between the two scanning systems [68,69].

In detail, the Italian sample from the Frassetto collection was CT scanned with a 64-slice Brilliance, Philips Medical System, Eindhoven-the Netherlands at the Department of Diagnostic Imaging of Santa Maria delle Croci Hospital in Ravenna (Italy), with the following relevant parameter setting: 140kVp, a tube exposure time of 1645 ms, a slice thickness of 1.00mm, filter type YD, Reconstruction Field of View (FOV) of 500 mm for Sassari sample and 343 mm for Bologna sample. Subsequently, the raw data were reconstructed as 16-bit unsigned DICOM images using the following voxel sizes: 1) Bologna sample: 0.960 x 0.960 x 0.7 mm; 2) Sassari sample: 0.976 x 0.976 x 0.5 mm. The CT image data were segmented in Avizo 7.1 (Visualization Science Group Inc.) to obtain 3D digital models of each talus. The tali from New York were digitally acquired using a Konica Minolta Vivid 910 surface laser scanner (X: ± 0.22 mm, Y: ± 0.16 mm, Z: ± 0.10 mm). Surface scan data were processed (i.e., mesh alignment, cleaning) using the scanner’s associated software (Polygon Editing Tool, Konica Minolta, 2006) and Geomagic Studio 8 (3D Systems). In each case the 3D models were saved in STL format and loaded into Viewbox 4 (dHAL Software) for landmarking.

### Geometric morphometrics and statistical analysis

**Fig 1:**
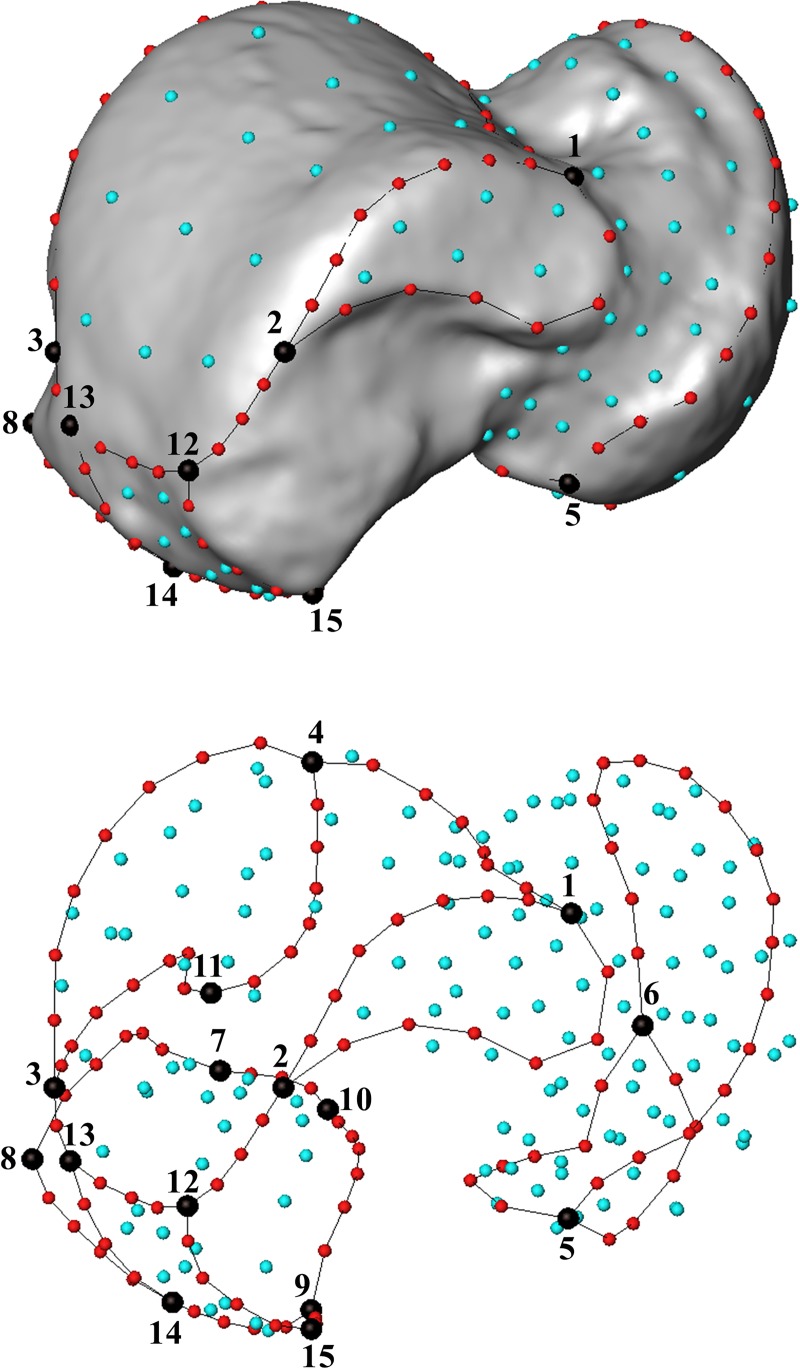
Template with landmarks (black), curve and surface semilandmarks (red and light blue, respectively) digitized on a left talus. See Table 1 for a detailed description of the anatomical landmarks.

A 3D template of 251 (semi)landmarks (15 anatomical landmarks, 105 curve semilandmarks and 131 surface semilandmarks) was created in Viewbox 4 software (Fig 1 and Table 1) and subsequently applied to the targets. The semilandmarks were allowed to slide on the curves (curves semilandmarks) and on the surface (surface semilandmarks) to minimize the thin-plate spline (TPS) bending energy between the target and the template [46,59]. Semilandmarks are not anatomical landmarks but when a TPS interpolation function is applied they became geometrically homologous between individuals, thus allowing for analysis in conjunction with traditional landmarks [58,59].

**Table 1:** List of anatomical landmarks of the template for the GM analysis of the talus. Type of landmarks according to Bookstein [73].

Landmarks	Type^1^	Labels
Most distal lateral point of contact between the medial malleolar facet and the trochlear surface	II	1
Most proximal point of contact between the medial malleolar facet and the trochlear surface	II	2
Most proximal point of contact between the lateral malleolar facet and the trochlear surface	II	3
Most distal point of contact between the lateral malleolar facet and the trochlear surface	II	4
Most medial point of contact on the head/navicular facet	III	5
Most lateral point on the head/navicular facet	III	6
Most lateral point on the proximal calcaneal facet	III	7
Deepest (most dorsal) point on the proximal calcaneal facet	III	8
Most proximo-medial point on the proximal calcaneal facet	III	9
Most disto-lateral point on the proximal calcaneal facet	II	10
Most plantar point on the lateral malleolar facet	III	11
Flexor hallucis longus: most distal point on the medial margin	III	12
Flexor hallucis longus: most distal point on the lateral margin	III	13
Flexor hallucis longus: intersection with calcaneus curve	II	14
Flexor hallucis longus: most postero-inferior prominent point	III	15

^1^ Type I, local (histological) points (e.g., meeting of structures, juxtapositions of tissues, etc.); Type II, geometrical homology points with equivalent biomechanical implications (e.g., point in a distinct margin between two articular facets, tooth tip,etc.); Type III, relative position on a feature (endpoints of maximum length, extremal points, etc.).

Landmarks and semilandmarks coordinates used to describe the specimens of the study are available in S1 Appendix, allowing the reproducibility of this research.

The (semi)landmark coordinates were allowed to slide against recursive updates of the Procrustes consensus and converted into shape coordinates, with scale, position and orientation standardized via Generalized Procrustes Analysis (GPA) [46,70] using the R package “geomorph” [71]. Size was measured as centroid size (CS), which is the square root of the summed squared distances between each (semi)landmark and the centroid of the (semi)landmark configuration [45,46].

A Principal Component Analysis (PCA) was performed on the Procrustes coordinates to explore the pattern of morphological variation across the sample. A form-space PCA (i.e., shape + size) was carried out by augmenting the Procrustes shape coordinates by the natural logarithm of CS, hereafter called lnCS [72]. Visualization of shape changes along the principal axes was obtained by TPS deformation [73] of the Procrustes grand mean shape surface in Avizo 7.1 (Visualization Science Group Inc.).

Sex differences observed along the first two PCs (in both shape and form space) were statistically tested by ANalysis Of VAriance (ANOVA), which was also used for CS within each population and for the pooled sample. To support the ANOVA results, the effect size (Cohen’s d) and a power analysis were performed to identify the minimum sample size required to test the null hypothesis that males and females have significantly different means (p < 0.05) [74].

Shape variation related to size (static allometry) was investigated by Pearson product-moment correlation coefficients (r) of shape variables (PCs) against lnCS. Then, multivariate regression of shape and form variables (using all the PCs) on lnCS was carried out to compute the intragroup allometric trajectory across the talar female-male morphospace. A permutation test (N = 1000) on lengths (i.e., magnitude of the variability) and angles between group’s trajectories was performed to assess whether the amount of sexual dimorphism differs significantly (i.e., P<0.05) among populations [75]. For each permutation test, specimens were randomly reassigned with respect to groups (i.e., Sassari, Bologna and New York), and new trajectories were computed.

Finally, we used ‘leave-one-out’ cross validation linear discriminant analysis (LDA) of shape space PC scores, form space PC scores, and CS alone to classify the specimens (i.e., either male or female). The number of PCs used for LDA varied for each analysis in order to find the minimum optimal combination of variables within the first 10 PCs (deemed relevant based on the cut-off of 70% of variation proposed by Jolliffe [76]). Data were processed and analyzed in R v. 3.5.1 (R Development Core Team, 2018).

### Reconstruction of fragmentary talus

**Fig 2:**
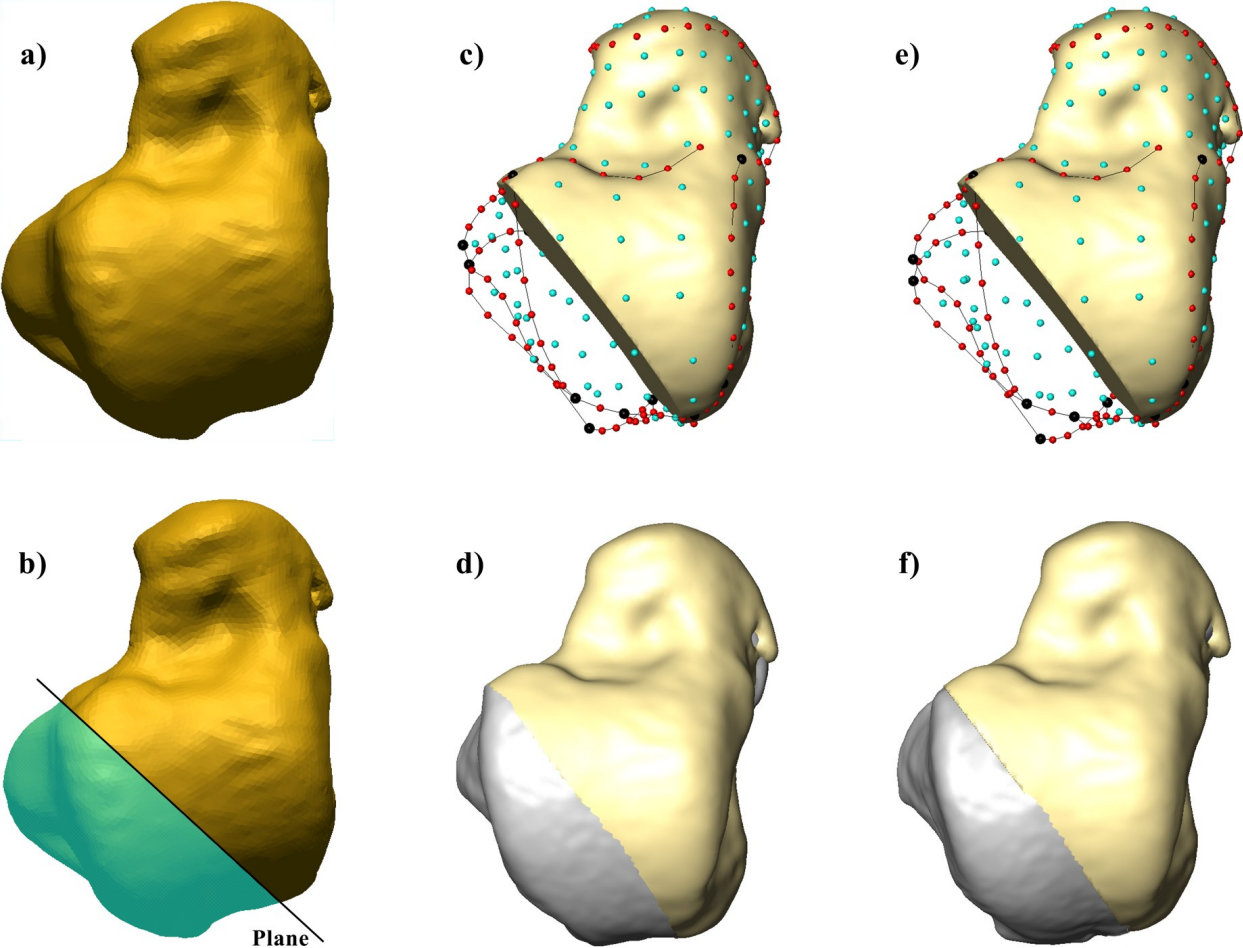
The left talus of BO-F-45 individual of Bologna (a) and the cutting plane used for the virtual resection (resected area in light blue) (b). Estimation of (semi)landmarks and reconstruction of the missing portion (in gray) based on the mean of both the Bologna sample (c, d) and the pooled sample (e, f).

In order to assess the utility of GM in cases where virtual reconstruction is necessary, we selected a talus of a female individual from the Bologna sample (BO-F-45) that was damaged at the talar head (Fig 2A). In this case the damage would make it impossible for linear measurements to be taken that are frequently used for discriminant function equations [35,36,77], resulting in the exclusion of the specimen from sex assessment [35]. Here the damage was exaggerated, whereby a more extensive “digital fracture” was created by resecting a portion of the talus (i.e., from the most lateral anterior margin of the trochlea to the mid of the flexor hallucis longus groove) using a cutting plane in Geomagic Design X (3D System) (Fig 2B). The missing portions were then estimated using morphological information from reference specimens by means of the TPS interpolation function in Viewbox 4 software [59,62]. Since the Procrustes mean shape is an effective reference for the reconstruction of missing portions [78], two reconstructions were tested based on two different reference specimens: 1) a reconstruction based on the mean configuration of the Bologna sample (Fig 2C and 2D), which represents the ideal condition due to the provenance of the case study; 2) a reconstruction based on the mean of the pooled sample (Fig 2E and 2F), which might represent an alternative solution in case the population’s provenance of the case study is unknown. In both cases, the virtual reconstruction of the talus was undertaken by estimating the position of the (semi)landmarks that fall in the missing regions (Fig 2C and 2E). The virtually reconstructed tali were then projected in the form-space PCA previously computed (i.e., the reconstruction by the mean of the Bologna sample in the Bologna form-space PCA; the reconstruction by the mean of the pooled sample in the pooled form-space PCA), and sex was predicted using the discriminant functions obtained for both the pooled and Bologna samples.

## Results

### Interspecific population sex assessment

**Fig 3:**
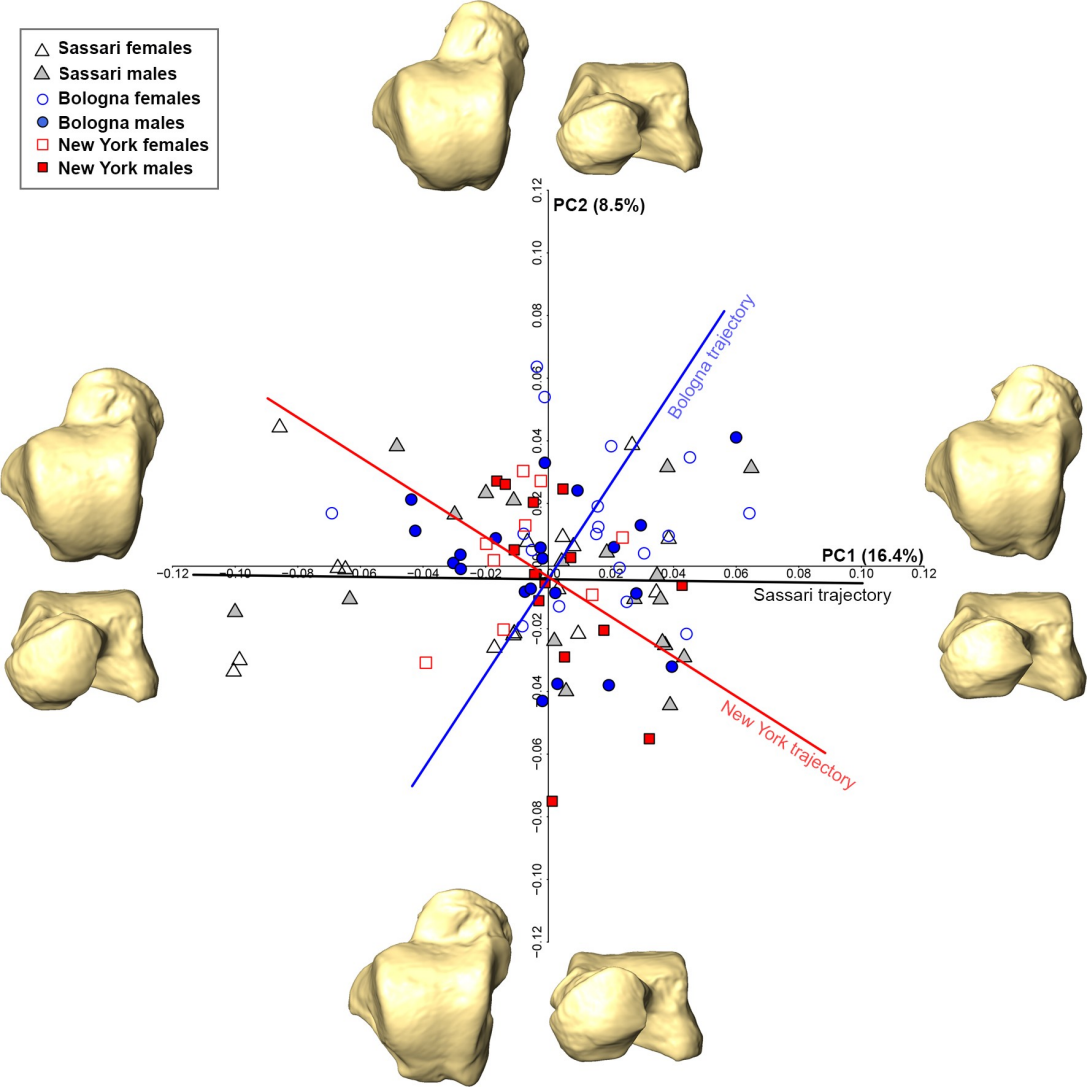
Shape space PCA plot of the pooled sample and shape warps along axes. Sassari individuals are in black, Bologna individuals in blue and New York individuals in red. Intragroup allometric trajectory (black for Sassari, blue for Bologna and red for New York) are shown in the PCA plot. The deformed mean tali in the four directions of the PCs are drawn at the extremity of each axis.

The shape-space PCA plot of the pooled sample shows a considerable degree of overlap among individuals (Fig 3). The first two PCs account for 24.9% of the total variance and do not contribute to separating males from females (ANOVA; PC1: Df = 1, *F*-test = 1.047, *P* = 0.30; PC2: Df = 1, *F*-test = 2.162, *P* = 0.14). A sample size of 98 achieved 82.2% power using PC1 scores and 98.6% power using PC2 scores for detecting a small effect size (PC1, -0.207; PC2, 0.298) with a significance level of 0.05. Subtle morphological differences are observed in the extreme shape of the PC1 and PC2 axes, in particular the talar head is more expanded on negative PC1 and negative PC2 and reduced on positive PC1 and positive PC2. A Pearson’s correlation test shows that PC1 is correlated with lnCS (r = 0.25; P<0.05), i.e., static allometry could account for morphological differences along this axis.

Permutation tests show that angles between group trajectories differ significantly between Sassari and Bologna (α = 103.5°, P<0.01), as well as New York and Bologna (α = 97.3°, P<0.05), but not between Sassari and New York (α = 57.8°, P = 0.36). No differences in length are observed among the allometric trajectories.

**Fig 4:**
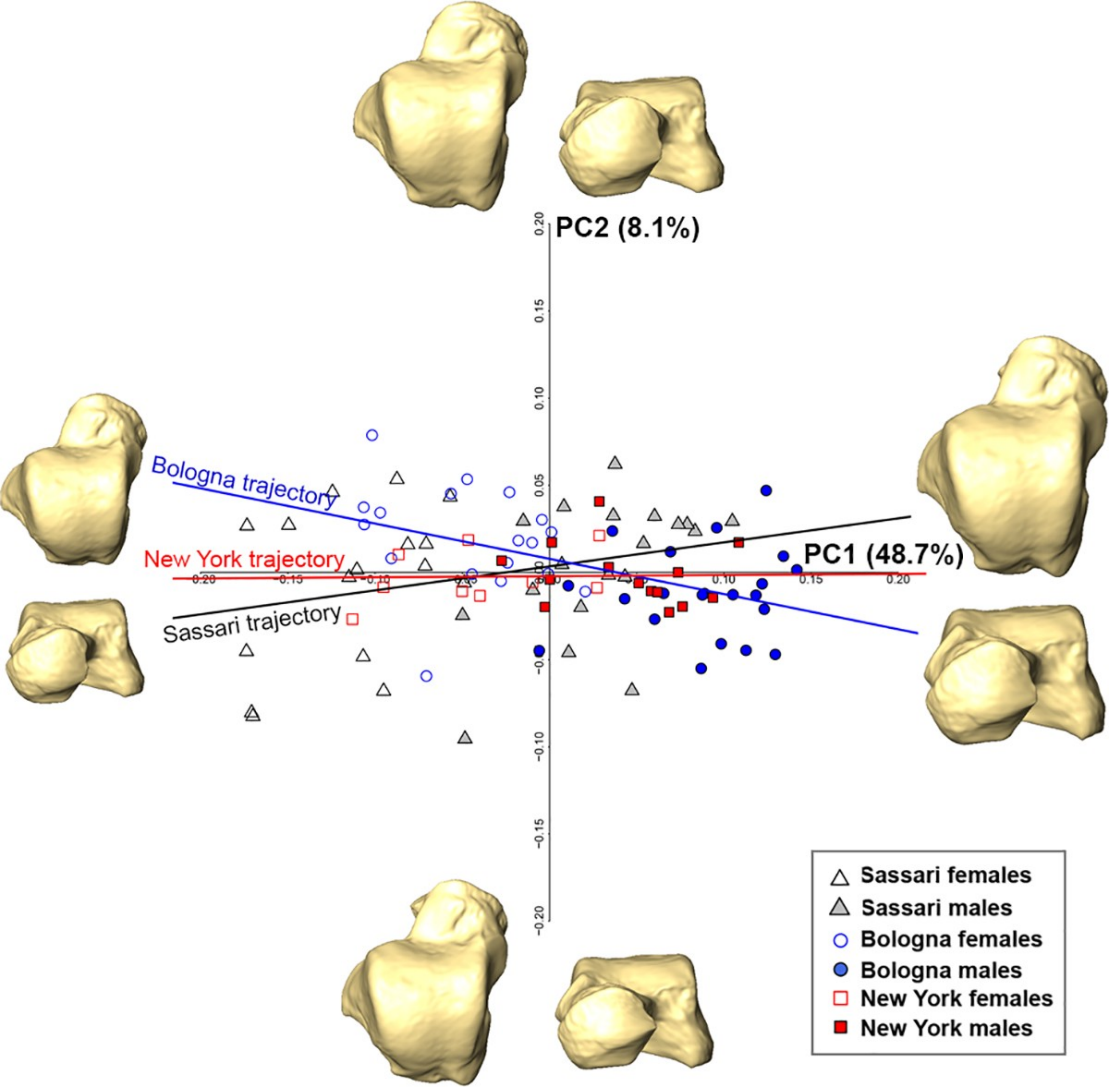
Form space PCA plot of the pooled sample and shape warps along axes. Sassari individuals are in black, Bologna individuals in blue and New York individuals in red. Intragroup allometric trajectory (black for Sassari, blue for Bologna and red for New York) are shown in the PCA plot. The deformed mean tali in the four directions of the PCs are drawn at the extremity of each axis.

Form space PC1 (48.7%), which retains all size information (r = 0.99; P<0.001), significantly separates males and females (ANOVA; Df = 1, *F*-test = 147.5, P<0.001) with 100% power to detect an effect size of -2.466 (P<0.05), while PC2 (8.1%) does not account for differences among sexes (ANOVA; Df = 1, *F*-test = 1.889, P = 0.17; 97.3% power with an effect size of 0.279) and is not correlated with lnCS (r = -0.05; P = 0.6) (Fig 4). Positive PC1 accounts for the relative superior-inferior lateral expansion of the talar head and more concave medial malleolar facet (i.e., male shape), while negative PC1 is related to a more rectangular and horizontally-inclined talar head, as well as a less concave medial malleolar facet (i.e., female shape). Allometric trajectories are significantly different between Sassari and Bologna (α = 23.7°, P<0.01), as well as New York and Bologna (α = 20.8°, P<0.05), but not between Sassari and New York (α = 15.9°, P = 0.53). Furthermore, the magnitude of the intergroup allometric variation distinguishes Sassari from Bologna (P<0.05).

Finally, the ANOVA shows that CS is significantly different between males and females (ANOVA; Df = 1, *F*-test = 151.7, P<0.001), achieving 100% power with a large effect size of -2.501.

Cross-validation LDA of the pooled sample is highly accurate when using the first 6 form space PCA scores (91.8%) and CS (87.7%). The number of correctly classified individuals drops when using shape space PCs, with accuracy reaching 66.3% using 9 PCs (Table 2).

**Table 2:** Accuracy of classification using shape, form variables and centroid size of each population and pooled sample.

* *	Predicted group membership				
	Male		Female		Total
	N	%	N	%	%
*Sassari*					
6 shape-space PCs	15/19	78.9	15/17	88.2	83.3
Centroid size	17/19	89.5	17/17	100	94.4
2 form-space PCs	18/19	94.7	17/17	100	97.2
*Bologna*					
7 shape-space PCs	16/21	76.2	15/18	83.3	79.4
Centroid size	19/21	90.5	18/18	100	94.9
1 form-space PCs	19/21	90.5	17/18	94.4	92.3
*New York*					
7 shape-space PCs	11/14	78.6	8/9	88.9	82.6
Centroid size	10/14	71.4	7/9	77.8	73.9
1 form-space PCs	11/14	78.6	7/9	77.8	78.2
*Pooled sample*					
9 shape-space PCs	37/54	68.5	28/44	63.6	66.3
Centroid size	47/54	87	39/44	88.6	87.7
6 form-space PCs	50/54	92.6	40/44	90.9	91.8

### Intraspecific population sex assessment

**Fig 5:**
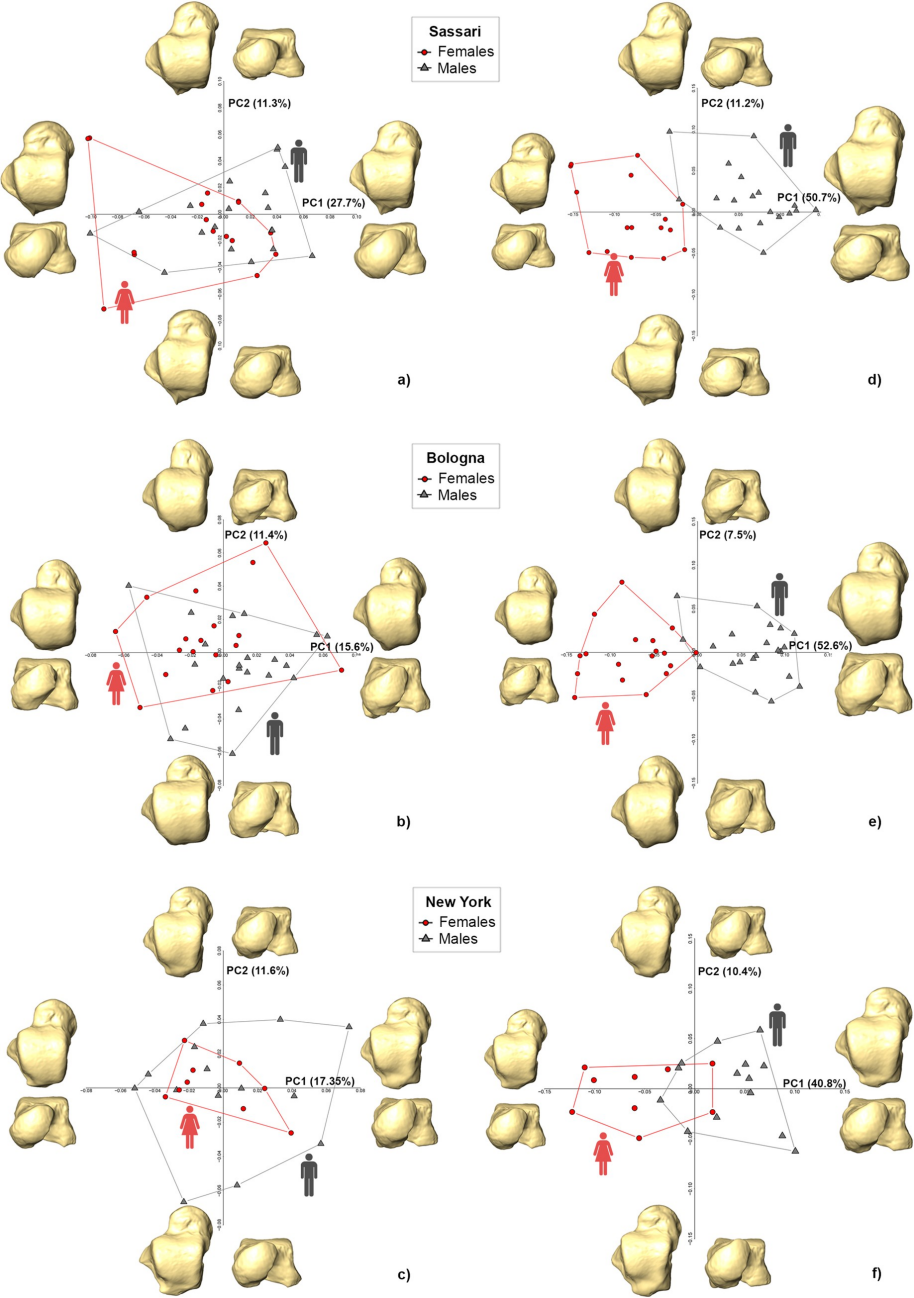
Shape (left) and form (right) space PCA plots for Sassari (a and d), Bologna (b and e) and New York (c and f). The deformed mean tali in the four directions of the PCs are drawn at the extremity of each axis.

Fig 5 shows the PCA plots in both shape and form space for each modern human population and the relative shape changes along the PC axes.

Overall, results in the shape space PCA suggest that there are no significant differences (P>0.05) driven by sexual dimorphism in the talar shape of the populations considered in this study (Fig 5A, 5B and 5C). This result is confirmed by the low discriminant accuracy, which ranges from 79.4% to 83.3% (Table 2). Power analysis reveals that Sassari and Bologna both have 99.9% power using PC1 scores with medium effect size (-0.647 for Sassari and -0.602 for Bologna), while New York achieved just 27% power to detect an effect size of -0.205 using an ANOVA with a significance level of 0.05. The effect size for PC2 scores is negligible for Sassari (-0.18) and New York (0.274) with a power of 32.5% and 6.0% respectively, while in Bologna the effect size is medium (0.628) with a power of 99.9%.

In form space PCA of the Sassari sample (Fig 5D), PC1 (50.7%) is strongly correlated with lnCS (r = 0.98; P <0.001) and significantly segregates males from females (ANOVA; Df = 1, *F*-test = 80.17, P<0.001; 100% power with an effect size of -2.989). Negative PC1 (i.e., Sassari females) possesses a shorter talar neck, with a medio-laterally extended navicular facet (from dorsal view), as well as a less concave and less anteriorly extended medial malleolar facet, compared with positive PC1 (i.e., Sassari males). DFA on the first two form space PCs returns the highest accuracy (97.2%) found in this study (Table 2). The importance of size is further supported by significant differences in CS (ANOVA; Df = 1, *F*-test = 97.31, P<0.001; 100% power with an effect size of -3.293), bringing the accuracy to 94.4%.

Similarly, form space PC1 (52.6%) significantly separates males and females of the Bologna sample (ANOVA; Df = 1, *F*-test = 105.6, P<0.001; 100% power with an effect size of 3.3) (Fig 5E). However, because no relevant allometric shape changes are recognized along the PC1 axis, the separation is largely driven by size (r = -0.99; P <0.001). DFA on the first form space PCs can correctly discriminate 92.3% of the individuals, while incorporating CS (ANOVA; Df = 1, *F*-test = 101.2, P<0.001; 100% power with an effect size of -3.23) brings the accuracy to 94.9% (Table 2).

Similarly, in form-space PCA males and females of the New York sample (Fig 5F) are significantly different along PC1 (40.8%; ANOVA, Df = 1, *F*-test = 22.1, P<0.001; 100% power with an effect size of -2.012), even though the two groups overlap in the middle of the plot. As for the Bologna sample, the separation is mainly driven by size (r = 0.99; P <0.001). Sex differences are significant using CS (ANOVA; Df = 1, *F*-test = 20.2, P<0.001; 100% power with an effect size of -1.921). Accuracy of the LDA is higher using the first form space PC scores (78.2%) than CS (73.9%), and in both cases are lower than the values obtained for the other two populations.

### Sex assessment of virtually reconstructed talus

**Fig 6:**
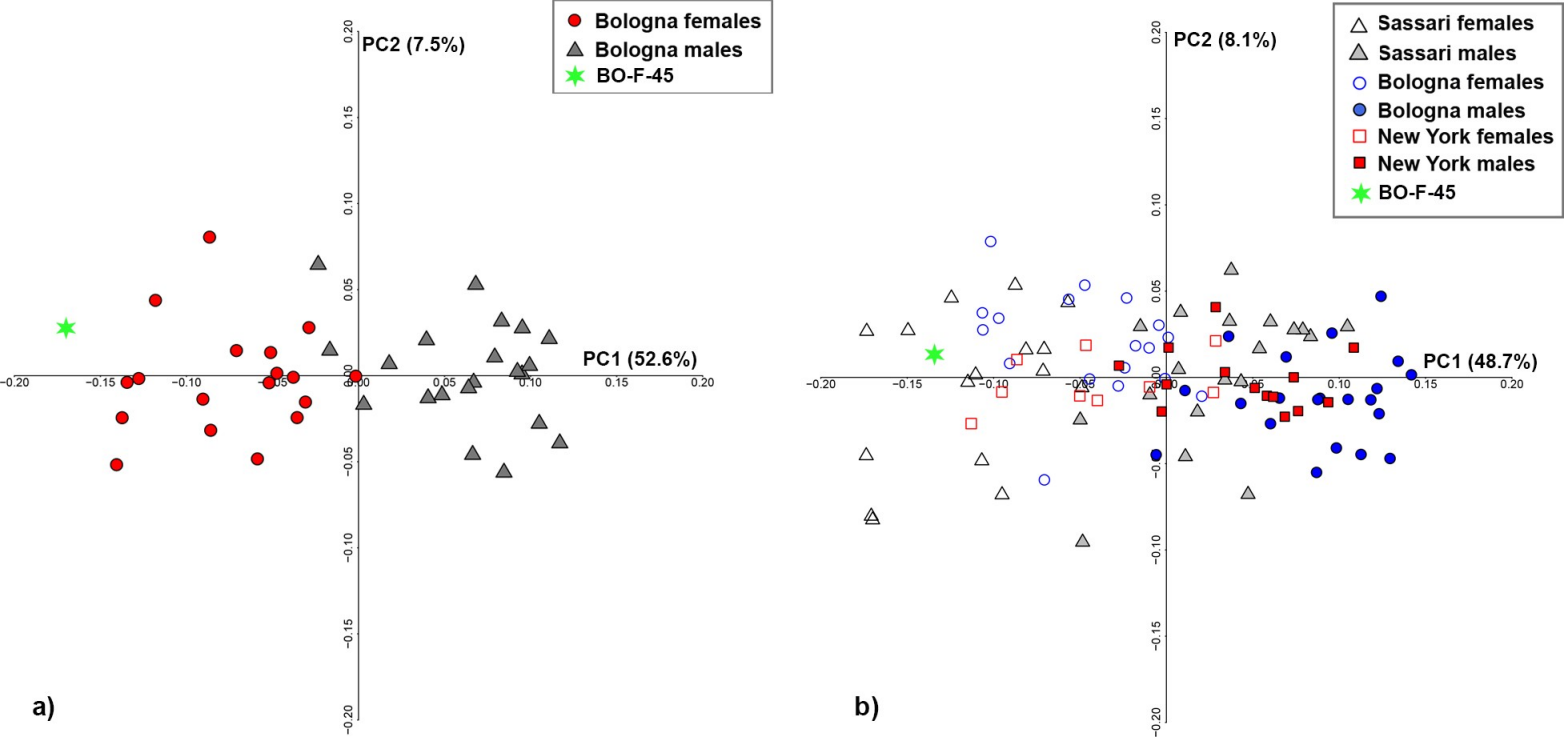
Form space PCA plots of the Bologna sample (a) and pooled sample (b). The green star represents the BO-F-45 talus reconstructed based on the Bologna sample mean (projected in PCA plot of Bologna displayed in a) and the pooled sample mean (projected in PCA plot of pooled sample displayed in b).

The two digitally reconstructed tali were projected into the form space PCA plot computed for the Bologna (Fig 6A) and pooled samples (Fig 6B), respectively. In both cases, specimen BO-F-45 falls close to the female group.

Indeed, the BO-F-45 talus was correctly classified as female (_Ppost_ = 100%) using either 6 form space PCs or CS of the pooled sample. Similarly, the first form space PCs and CS of the Bologna sample predict the sex of this individual as female with a probability of 99.9%.

## Discussion

Human foot bones are often retrieved as isolated elements in both archaeological and forensic contexts and consequently they have been the focus of several scientific contributions for sex determination and stature and age estimation [24,33,34,49–51,79–82]. Indeed, it is broadly accepted that the talus and the calcaneus are good indicators of biological sex, due to the weight bearing function of the foot and the resulting size differences [17,33,35–41,77]. In this study, we applied geometric morphometrics techniques to tali to investigate the role of shape, form, and size in determining biological sex. We analyzed samples representing three modern human populations from the early 20^th^ century (Sassari, Bologna, New York) issuing from different geographical locations (Italy and USA). We followed two different approaches, i.e., 1) considering the combined populations as a unique sample where we explored sex-related interpopulation trajectories and 2) focusing on each individual population to assess the discriminatory power of the talus for sex determination. Finally, we show that GM methods can aid in the reconstruction of fragmentary tali, ultimately overcoming the limits of traditional approaches utilizing linear measurements [17,35–37,39,40,43,77].

When considering the pooled early 20^th^ century sample, we found that sexual dimorphism differs significantly among populations (Figs 3 and 4). Indeed, a permutation test found a significant difference for intragroup allometric trajectories between Sassari and Bologna, as well as New York and Bologna in both shape and form space. However, this is not the case between Sassari and New York. Taken together this suggests that a population-specific approach should be used to evaluate sexual dimorphism in modern human tali. In the form space PCs of the pooled sample, individuals were correctly classified ~92% of the time, ultimately emphasizing the crucial role of size for sex determination based on the talus. The same holds when turning to a specific population approach, where either form space PCs or CS provide the best outcome, despite differences in the accuracy of the results among the populations.

Overall, our results are in agreement with those of Gualdi-Russo [35] relative to one of the populations we analyzed (i.e., Bologna), who demonstrated that male individuals exhibit larger talar measurements than females. Here, to the exclusion of fragmentary tali, our results supports the use of traditional linear measurements for sex determination of the tali [17,35–37,39,40,43,77].

Even so, our study based on a 3D GM adds something more to the current debate on sex-related talar morphometric changes that may not be evident with traditional analytical methods. Specifically, here we found allometric differences between males and females from the Sassari sample, where increasing size led to the talar neck becoming longer, the head less mediolaterally extended, and the medial malleolar facet more concave and anteriorly extended. This was not observed in the other populations and, while these slight morphological differences may not reflect adaptions given identical locomotion, it may reflect variation in footwear and lifestyle (e.g., posture, gait kinematics, joint angles, nutrition, daily activity patterns) [37,48–50,52–54,83].

Finally, we also must emphasize the utility offered by the (semi)landmark-based approach employed here, where we showed its capacity to deal with fragmentary talar remains that may otherwise be excluded from forensic analysis. In this case we digitally exaggerated a fracture that would nullify a set of traditional linear measurements (e.g., length and width of the talus, length and breadth of the trochlea, length and breadth of the posterior articular surface for the calcaneus), and then carried out two digital reconstructions based on the mean of both the pooled sample and the sample it issued from (Bologna). Being that our results from the known sample indicated that the shape differences between the sexes are minimal and closely related to allometry, it is not surprising that the TPS interpolation scaled the reference specimen into the target (i.e., the fragmentary talus). Despite this, both virtual reconstructions were found to be very similar, allowing for the correct classification of sex with a P_post_ between 99.9% (Bologna mean) and 100% (pooled mean). This suggests that, although it is desirable to select a reference specimen from the population that matches that of the target (i.e., case study), it is still possible to obtain a favorable reconstruction and classification based on a pooled mean. Still, further studies are needed to create a reference dataset of fragmentary tali that may be used to validate the results presented here.

In conclusion, the results of this study confirm that the talus is a good indicator of sexual dimorphism and that it can be used in forensic scenarios where only isolated human remains are recovered (e.g., mass disasters, commingled burials, altered taphonomic contexts). Furthermore, considering the poor preservation/fragmentary nature of bones retrieved in forensic/bioarchaeological contexts, we suggest that a 3D GM-based virtual reconstruction, similar to that performed here, may be of use to researchers wishing to include sex estimation from remains that would otherwise be removed from such a fundamental portion of analysis.

## Supporting information

S1 Appendix 3D coordinates of the landmarks and semi-landmarks of the sample used in the study. (XLSX)
